# Discovering Functional DNA Elements Using Population Genomic Information: A Proof of Concept Using Human mtDNA

**DOI:** 10.1093/gbe/evu116

**Published:** 2014-06-09

**Authors:** Daniel R. Schrider, Andrew D. Kern

**Affiliations:** Department of Genetics, Rutgers, The State University of New Jersey

**Keywords:** population genetics, natural selection, mitochondria

## Abstract

Identifying the complete set of functional elements within the human genome would be a windfall for multiple areas of biological research including medicine, molecular biology, and evolution. Complete knowledge of function would aid in the prioritization of loci when searching for the genetic bases of disease or adaptive phenotypes. Because mutations that disrupt function are disfavored by natural selection, purifying selection leaves a detectable signature within functional elements; accordingly, this signal has been exploited for over a decade through the use of genomic comparisons of distantly related species. While this is so, the functional complement of the genome changes extensively across time and between lineages; therefore, evidence of the current action of purifying selection in humans is essential. Because the removal of deleterious mutations by natural selection also reduces within-species genetic diversity within functional loci, dense population genetic data have the potential to reveal genomic elements that are currently functional. Here, we assess the potential of this approach by examining an ultradeep sample of human mitochondrial genomes (*n* = 16,411). We show that the high density of polymorphism in this data set precisely delineates regions experiencing purifying selection. Furthermore, we show that the number of segregating alleles at a site is strongly correlated with its divergence across species after accounting for known mutational biases in human mitochondrial DNA (*ρ* = 0.51; *P* < 2.2 × 10^−16^). These two measures track one another at a remarkably fine scale across many loci—a correlation that is purely the result of natural selection. Our results demonstrate that genetic variation has the potential to reveal with surprising precision which regions in the genome are currently performing important functions and likely to have deleterious fitness effects when mutated. As more complete human genomes are sequenced, similar power to reveal purifying selection may be achievable in the human nuclear genome.

## Introduction

Only 1–2% of human genome lies within protein-coding sequence (Lander et al. 2001). Determining the extent to which the remainder of the genome is functional is crucial to our understanding of human biology. A variety of recently developed experimental techniques have aided in the search of noncoding DNA for functional elements (Dunham et al. 2012); however, on their own these techniques can produce a huge number of false positives (Graur et al. 2013). Searches for the evolutionary signature of purifying selection have therefore proved a more fruitful strategy for identifying functional elements; indeed phylogenetic searches comparing sequences of related species have revealed that approximately 5% of the human genome is constrained by natural selection (Chinwalla et al. 2002; Siepel et al. 2005; Lunter et al. 2006; Birney et al. 2007; Davydov et al. 2010), and similar strategies have been used to predict the phenotypic severity of mutations (Stone and Sidow 2005). Although whole-genome comparisons aimed at identifying the footprints of selection are highly effective, they have been used primarily to detect elements under constraint for hundreds of millions of years of evolutionary history (Siepel et al. 2005; Davydov et al. 2010). However, the set of functional elements in the genome experiences considerable turnover (Demuth et al. 2006). Comparative genomic techniques will fail in these instances, particularly for the supremely interesting cases of human-specific gain (Knowles and McLysaght 2009) and loss of function (Wang et al. 2006).

Surveys of genetic diversity within species, on the other hand, have the potential to identify regions currently experiencing purifying selection and that are therefore functional, as purifying selection will remove genetic diversity from such loci. Unfortunately, genetic variation in the human genome is quite sparse, with a comparison of any two homologous chromosomes uncovering less than 1 single-nucleotide polymorphism (SNP) every kilobase (Lander et al. 2001). Sampling more individuals, however, yields additional polymorphisms, and an ultradeep sample of mitochondrial variation from 16,411 genomes is available in the MITOMAP database (Ruiz-Pesini et al. 2007). These data are extremely polymorphic, with more than one SNP on every other base pair on average. This data set thus serves as an ideal proving ground for the approach of identifying functional constraint using massive amounts of polymorphism data, which will soon be available for nuclear genomes. Here, we show that the density of polymorphism in these data closely tracks divergence at a fine scale, implying that these data can indeed be used to reveal the strength of purifying selection in the human mitochondrial genome at a very high resolution. Our results suggest an enormous potential for population genomic data to uncover functional DNA elements, including those not conserved across species.

## Results and Discussion

**Fig. 1.—:**
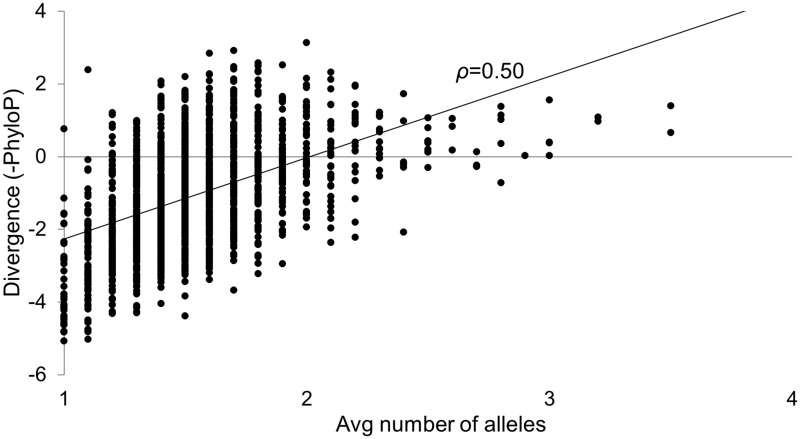
The correlation between polymorphism and divergence in the human mitochondrial genome. The average number of alleles per base pair in 10-bp windows is shown in the *x*-axis and divergence as measured by the negated phyloP score is shown in the *y*-axis.

We set out to determine the extent to which polymorphism data reveal the strength of purifying selection across the human mitochondrial genome and downloaded the coordinates of all 8,944 SNPs from MITOMAP (http://www.mitomap.org/MITOMAP, last accessed August 4, 2013). We reasoned that if the density of polymorphism was governed by the amount of purifying selection acting on each site, then SNP density would be correlated with divergence across species, in accordance with expectations under the Neutral model (Kimura 1982). This is indeed what we observe in the form of a strong correlation between the number of alleles per site and its average negated phyloP score (Siepel et al. 2006) measuring divergence across vertebrates (Spearman’s *ρ* = 0.52; *P* < 2.2 × 10^−16^). This correlation is also highly significant when averaging polymorphism and divergence within 10-bp adjacent windows (*ρ* = 0.50; *P* < 2.2 × 10^−16^; fig. 1).

Although this observation is consistent with purifying selection both removing diversity and constraining divergence at functional elements, such a pattern could also be generated by variation in the spontaneous mutation rate. It has been shown that mutation rate in the mitochondria varies according to the duration for which a given site remains single stranded on the H strand (*D*_ssH_) during DNA replication (Reyes et al. 1998). We also find evidence for this in the form of a significant correlation between divergence at each site and the duration the site is single stranded on the H strand during replication, although this correlation is far weaker than that shared between polymorphism and divergence (*ρ* = 0.11; *P* < 2.2 × 10^−16^). Moreover, after correcting for *D*_ssH_, the correlation between polymorphism and divergence at individual sites is essentially unchanged and still highly significant (*ρ* = 0.49; *P* < 2.2 × 10^−16^).

**Fig. 2.—:**
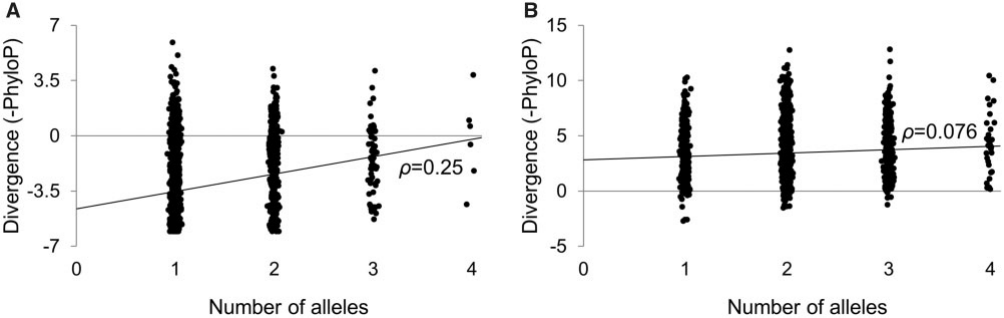
The correlation between polymorphism and divergence at nonsynonymous and synonymous sites. The number of alleles observed at each site is shown in the *x*-axis, and divergence (negative phyloP score) is shown in the *y*-axis at (*A*) second codon position (nonsynonymous) sites and (*B*) 4-fold degenerate (synonymous) sites. We added noise to the number of alleles to reveal the density of sites along the *y*-axis.

Rather than being driven by a subset of mitochondrial loci, this correlation is significant (at *P* < 0.05) in 36/37 genes and is significant in 35/37 genes after correcting for *D*_ssH_ (table 1). Similarly, polymorphism and divergence are more strongly correlated in protein-coding (*ρ* = −0.53; *P* < 2.2 × 10^−16^) and RNA-coding genes (*ρ* = −0.43; *P* < 2.2 × 10^−16^) than noncoding DNA within the control region (*ρ* = −0.23; *P* = 9.3 × 10^−15^) or outside of it (*ρ* = −0.30; *P* = 0.021). This correlation is also far stronger at nonsynonymous than synonymous sites (*ρ* = 0.25 for second codon position sites, *P* < 2.2 × 10^−16^; *ρ* = 0.079 for 4-fold degenerate sites, *P* = 3.6 × 10^−4^; fig. 2) as expected if purifying selection is a more predominant force at nonsynonymous sites. Finally, the minor allele frequencies of SNPs from the Human Mitochondrial Genome Database (mtDB; Ingman and Gyllensten 2006) are correlated with divergence (*ρ* = 0.076; *P* = 4.0 × 10^−16^), even though variation in mutation rate is not expect to affect allele frequencies. Thus, purifying selection uniquely drives patterns of polymorphism in the human mitochondrial genome. This finding supports previous reports that purifying selection is a prominent force in the mitochondrial genome (Rand and Kann 1996; Nielsen and Weinreich 1999; Elson et al. 2004; Stewart et al. 2008). The patterns we observed are not the result of positive selection, as the fixation of a beneficial mutation through a selective sweep removes all genetic diversity from a nonrecombining chromosome (Smith and Haigh 1974). We are thus limited to observing mutations occurring since the most recent sweep.

**Table 1:** Gene-Specific Correlations Between SNP Density and Negative phyloP Score

Gene Name	Gene Description	Gene Start (hg19)	Gene End (hg19)	Spearman’s *ρ*	*P*	Spearman's *ρ* (Correcting for *D*_ssH_)	*P*(Correcting for *D*_ssH_)
*MT-TF*	tRNA phenylalanine	579	649	−0.484	1.90 × 10^−5^	−0.468948	3.71 × 10^−5^
*MT-RNR1*	12S ribosomal RNA	650	1603	−0.429	<2.20 × 10^−16^	−0.4169046	<2.20 × 10^−16^
*MT-TV*	tRNA valine	1604	1672	−0.336	0.004783	−0.326022	0.006261
*MT-RNR2*	16S ribosomal RNA	1673	3230	−0.443	<2.20 × 10^−16^	−0.4435798	<2.20 × 10^−16^
*MT-TL1*	tRNA leucine 1	3231	3305	−0.301	0.008593	−0.29837	0.00932
*MT-ND1*	NADH dehydrogenase subunit 1	3308	4263	−0.542	<2.20 × 10^−16^	−0.5149936	<2.20 × 10^−16^
*MT-TI*	tRNA isoleucine	4264	4332	−0.269	0.02551	−0.2523745	0.03643
*MT-TQ*	tRNA glutamine	4330	4401	−0.463	4.19 × 10^−5^	−0.476385	2.34 × 10^−5^
*MT-TM*	tRNA methionine	4403	4470	−0.291	0.01611	−0.2880727	0.01721
*MT-ND2*	NADH dehydrogenase subunit 2	4471	5512	−0.516	<2.20 × 10^−16^	−0.461188	<2.20 × 10^−16^
*MT-TW*	tRNA tryptophan	5513	5580	−0.509	9.42E × 10^−6^	−0.4626812	7.11 × 10^−5^
*MT-TA*	tRNA alanine	5588	5656	−0.429	0.0002381	−0.4412621	0.0001475
*MT-TN*	tRNA asparagine	5658	5730	−0.378	0.0009899	−0.3765626	0.001025
*MT-TC*	tRNA cysteine	5762	5827	−0.596	1.26 × 10^−7^	−0.5709837	5.55 × 10^−7^
*MT-TY*	tRNA tyrosine	5827	5892	−0.352	0.00371	−0.3486535	0.004118
*MT-CO1*	Cytochrome *c* oxidase subunit I	5905	7446	−0.633	<2.20 × 10^−16^	−0.6186776	<2.20 × 10^−16^
*MT-TS1*	tRNA serine 1	7447	7515	−0.627	8.31 × 10^−9^	−0.6107229	2.51 × 10^−8^
*MT-TD*	tRNA aspartic acid	7519	7586	−0.365	0.002186	−0.320253	0.007758
*MT-CO2*	Cytochrome *c* oxidase subunit II	7587	8270	−0.573	<2.20 × 10^−16^	−0.5291891	<2.20 × 10^−16^
*MT-TK*	tRNA lysine	8296	8365	−0.331	0.005158	−0.2809919	0.01846
*MT-ATP8*	ATP synthase F0 subunit 8	8367	8573	−0.266	0.0001042	−0.2637231	0.0001233
*MT-ATP6*	ATP synthase F0 subunit 6	8528	9208	−0.389	<2.20 × 10^−16^	−0.3823366	<2.20 × 10^−16^
*MT-CO3*	Cytochrome *c* oxidase subunit III	9208	9991	−0.538	<2.20 × 10^−16^	−0.5314281	<2.20 × 10^−16^
*MT-TG*	tRNA glycine	9992	10059	−0.396	0.0008191	−0.3404812	0.004497
*MT-ND3*	NADH dehydrogenase subunit 3	10060	10405	−0.510	<2.20 × 10^−16^	−0.5000739	<2.20 × 10^−16^
*MT-TR*	tRNA arginine	10406	10470	−0.470	7.66 × 10^−5^	−0.4415206	0.0002317
*MT-ND4L*	NADH dehydrogenase subunit 4L	10471	10767	−0.491	<2.20 × 10^−16^	−0.5133884	<2.20 × 10^−16^
*MT-ND4*	NADH dehydrogenase subunit 4	10761	12138	−0.561	<2.20 × 10^−16^	−0.5419698	<2.20 × 10^−16^
*MT-TH*	tRNA histidine	12139	12207	−0.277	0.02112	−0.1786413	0.1419
*MT-TS2*	tRNA serine 2	12208	12266	−0.470	0.0001712	−0.489146	8.45 × 10^−5^
*MT-TL2*	tRNA leucine 2	12267	12337	−0.374	0.001311	−0.3543069	0.002434
*MT-ND5*	NADH dehydrogenase subunit 5	12338	14149	−0.532	<2.20 × 10^−16^	−0.5141183	<2.20 × 10^−16^
*MT-ND6*	NADH dehydrogenase subunit 6	14150	14674	−0.500	<2.20 × 10^−16^	−0.4776221	<2.20 × 10^−16^
*MT-TE*	tRNA glutamic acid	14675	14743	−0.347	0.003468	−0.3476731	0.003421
*MT-CYB*	Cytochrome *b*	14748	15888	−0.551	<2.20 × 10^−16^	−0.5025513	<2.20 × 10^−16^
*MT-TT*	tRNA threonine	15889	15954	−0.560	1.00 × 10^−6^	−0.4354723	0.0002578
*MT-TP*	tRNA proline	15957	16024	−0.103	0.4044	−0.1870294	0.1267

**Fig. 3.—:**
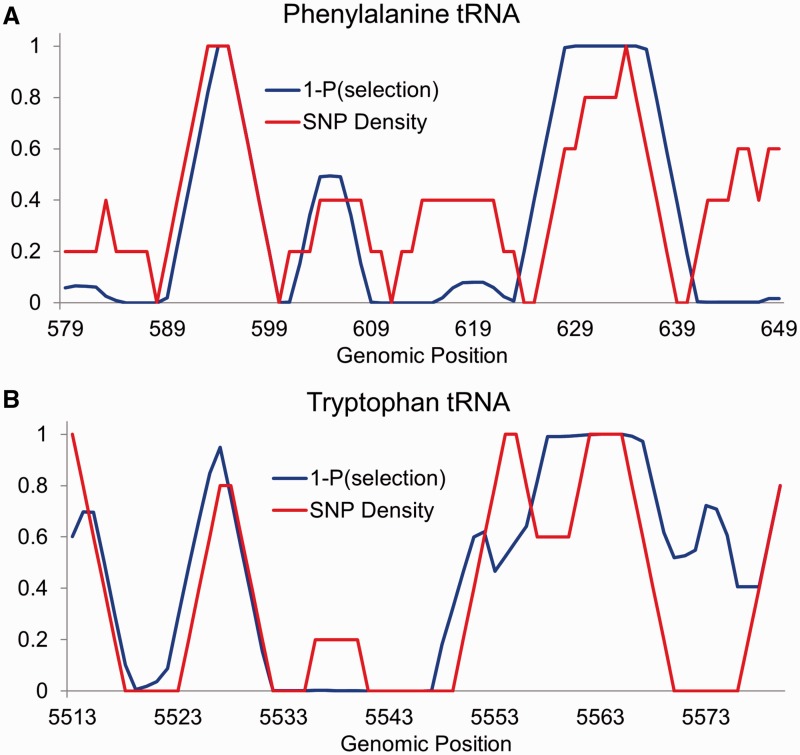
The probability of polymorphism versus the probability of unconstrained evolution across vertebrates. (*A*) The 5-bp sliding genomic windows showing SNP density (blue) and one minus the probability of conservation across vertebrates (red) according to phastCons (Siepel et al. 2005) across the phenylalanine tRNA gene. (*B*) The same plot for the tryptophan tRNA gene.

Having established that patterns of polymorphism across the human mitochondria are largely determined by purifying selection, we sought to determine the resolution at which these data reveal the strength of selection acting on particular sites in the genome. We examined patterns of SNP diversity and divergence in 5-bp sliding windows across each gene in the mitochondrial genome, observing the extent to which the two measures mirror one another on a small scale. In particular, within each window, we calculated the average SNP density per base pair and the average probability that the site is not conserved across vertebrates according to phastCons (Siepel et al. 2005). We find that for many loci, tRNA genes in particular, these two measures track one another to a surprising extent (e.g., the phenylalanine and tryptophan tRNA genes shown in fig. 3; the remaining tRNA genes are shown in supplementary fig. S1, Supplementary Material online). This result demonstrates that SNP density has the ability to reveal the strength of selection at a surprisingly detailed resolution—on the scale of a few base pairs.

**Fig. 4.—:**
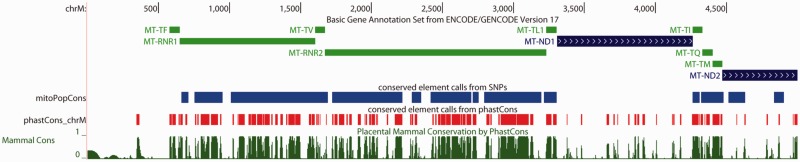
Comparison of conserved elements called from phylogenetic data and those called from population genetic data. This image form the UCSC Genome Browser shows positions 1–5,000 of the human mitochondrial genome. Conserved elements called from the polymorphism-based HMM (mitoPopCons) appear in blue, whereas phastCons elements obtained from a comparison of mammalian genomes appear in red. phastCons conservation probabilities are shown at the bottom in green. Gene locations are shown at the top.

As a simple proof of concept, we sought to use SNP density to predict function at a fine scale via a hidden Markov model (HMM; Rabiner 1989). Using a similar strategy as phastCons (Siepel et al. 2005), we learned a two-state HMM (constrained vs. unconstrained) where the observation for each site in the genome is the number of alleles at the site. We then used this HMM to predict constrained regions to which we refer as mitoPopCons elements. There is extremely strong overlap between mitoPopCons elements obtained from polymorphism data and phastCons elements predicted from divergence (*P* < 0.0001; fig. 4; see Materials and Methods). Although mitoPopCons recovers somewhat fewer genic base pairs than phastCons (35.4% of all genic base pairs are recovered by mitoPopCons versus 49.7% by phastCons), mitoPopCons elements contain fewer intergenic base pairs (0.75% of mitoPopCons base pairs are intergenic versus 3.4% of phastCons bases). Given the dramatically deeper evolutionary time period examined by phastCons data, that it seems to perform only marginally better than mitoPopCons underscores the potential of population genetic approaches. phastCons elements are smaller and more numerous (1,395 elements averaging 6.7 bp in length) than mitoPopCons elements (33 elements averaging 167 bp), perhaps implying that phylogenetic data allow for higher resolution prediction than even our dense polymorphism data. On the other hand, element length distributions may have been influenced by the difference in emission probability training methods used for mitoPopCons (trained via the Baum–Welch algorithm; see Materials and Methods) and phastCons elements. In any case, the success of this simple HMM shows that SNP diversity has the ability to accurately predict function at a fine scale in the human mitochondria.

We have shown that ultradense polymorphism data can be used to accurately detect functional nucleotides in the human mitochondrial genome, potentially at the level of the individual base pair, while sidestepping limitations of phylogenetic approaches. This result suggests that as whole-genome sequencing becomes more ubiquitous, it may become possible to perform such high-resolution prediction in the nuclear genome as well. Applying a polymorphism-based approach to the nuclear genome will present several additional challenges. First, independent assortment and recombination in the nuclear genome cause different loci to have distinct genealogical histories and therefore varying levels of diversity under neutrality, thereby potentially impeding the detection of selection. As another consequence of recombination, both positive (Smith and Haigh 1974) and negative selection (Charlesworth et al. 1993) will have localized effects on flanking variation, rather than genome wide as in the mitochondria. These forces will further increase variance in polymorphism at unselected sites and may thus obscure the signal of negative selection at selected sites.

Another difficulty of the nuclear genome is that its nucleotide diversity is far lower than that of the mitochondrial genome (Lander et al. 2001), meaning that an even larger number of sequences than examined here may be required to accurately detect selection. Moreover, the power to detect function increases logarithmically with sample size (supplementary fig. S2, Supplementary Material online). However, given the ever-increasing rate at which new human genome sequences are released, this problem may not be insurmountable. Finally, there is likely more variation in the strength of purifying selection acting in the nuclear genome than in the mitochondria. As a consequence, weakly constrained but still functionally important regions may evade detection, especially by a two-state method, allowing for only one level of constraint like the HMM used here.

If these hurdles can be overcome, approaches such as ours will then have an enormous impact on biological research, allowing for the discovery of the complete set of functional elements in the human genome and the degree to which new mutations at each site are deleterious. Such efforts will vastly improve predictions of the phenotypic impact of mutations occurring in humans and will prioritize searches for disease-causing mutations. This information will also reveal species-specific changes in selective pressures at the resolution of individual nucleotides, thereby greatly improving our understanding of how the functional components of genomes evolve.

## Materials and Methods

We converted all coordinates obtained from MITOMAP and mtDB from the Cambridge Reference Sequence (CRS) coordinate space to the human reference genome’s (GRCh37) coordinate space by aligning the two mitochondrial sequences using MUSCLE (Edgar 2004). We downloaded phyloP scores for each mitochondrial site from the UCSC Genome Browser Database (Meyer et al. 2013). Because there are no allele frequency data available for MITOMAP SNPs, we used the number of alleles at each site as our measure of diversity. For the correlation of minor allele frequency with divergence, we used frequencies of biallelic SNPs from mtDB. We measured the duration of single strandedness (*D*_ssH_) following Chong and Mueller (2013), using the coordinates of the light-strand origin of replication from MITOMAP. For the *D*_ssH_ analyses, we omitted sites within the control region. To control for the *D*_ssH_-divergence correlation, we fit a generalized linear model treating the number of segregating alleles at each site as a Poisson distributed response variable and with *D*_ssH_ value as the predictive variable using R. We then calculated the correlation between phyloP scores and the polymorphism residuals.

The HMM analysis was performed in MATLAB with the same transition matrix as used to learn the phastCons HMM for the UCSC Genome Browser (Kent et al. 2002). For each base pair, the emitted observation was the number of alleles (ranging one and four) found in the MITOMAP data set. The rationale for this approach was that after training the HMM, its two states should have different probabilities of emitting 1, 2, 3, or 4 alleles at a given site, and this would result in one state being more likely in stretches of DNA under selective constraint, whereas the other state would have higher likelihood in less constrained sequences. After assigning an initially random emission probability matrix, we then used Baum–Welch training to learn the emission parameters only for the constrained and unconstrained states by using large number of pseudocounts for transition matrix to ensure that it remained invariant during training. To guard against convergence at a poor local optimum, we repeated this process ten times and kept the emission matrix under which the observed sequence data had the highest likelihood. After training, we considered the state with the higher probability of emitting monomorphic sites to be the “functional” state and the other state to be “nonfunctional.” Next, we predicted constrained elements by using the Viterbi algorithm. We tested for significant overlap between our HMM predictions and mammalian phastCons elements from the UCSU Genome Browser by counting the number of base pairs constrained according to both methods and compared this count with those obtained after permuting the coordinates of our predictions. This was repeated for 10,000 permutations to obtain a *P* value.

To reveal the relationship between sample size and the HMM’s power to recover functional sites, we also repeated the HMM analysis as described above for different sample sizes using the number of alleles at each site in the mtDB set—the MITOMAP set could not be used for this as it lacks reliable allele frequency estimates. The sample sizes we examined ranged from 100 to 1,800, incrementing by 100. We also used a sample size of 1,864, which was the minimum sample size among all SNPs in the mtDB set. For each sample size, we randomly downsampled from the full mtDB data set by randomly drawing the desired number of alleles from the full set at each site without replacement. We then counted the number of distinct alleles observed at each site within the downsampled set, and this number was used as the observation for the HMM.

## Supplementary Material

Supplementary figures S1 and S2 are available at *Genome Biology and Evolution* online (http://www.gbe.oxfordjournals.org/).

Supplementary Data
