# Supplementary material for: Discovering Functional DNA Elements Using Population Genomic Information: A Proof of Concept Using Human mtDNA
Source: Genome Biol Evol. 2014 Jun 9;6(7):1542–8. doi: 10.1093/gbe/evu116 (PMC4122919; doi:10.1093/gbe/evu116)
Supplement: Supplementary Data [file supp_6_7_1542__index.html]

Discovering functional DNA elements using population genomic information: A proof of concept using human mtDNA — Discovering Functional DNA Elements Using Population Genomic Information: A Proof of Concept Using Human mtDNA — Supplementary Data 

# Discovering Functional DNA Elements Using Population Genomic Information: A Proof of Concept Using Human mtDNA

## Supplementary Data

files

**Files in this Data Supplement:**

- Supplementary Data - tif file
- Supplementary Data - tif file
