# Supplementary figures and images for: Discovering Functional DNA Elements Using Population Genomic Information: A Proof of Concept Using Human mtDNA
Source: Genome Biol Evol. 2014 Jun 9;6(7):1542–8. doi: 10.1093/gbe/evu116 (PMC4122919; doi:10.1093/gbe/evu116)

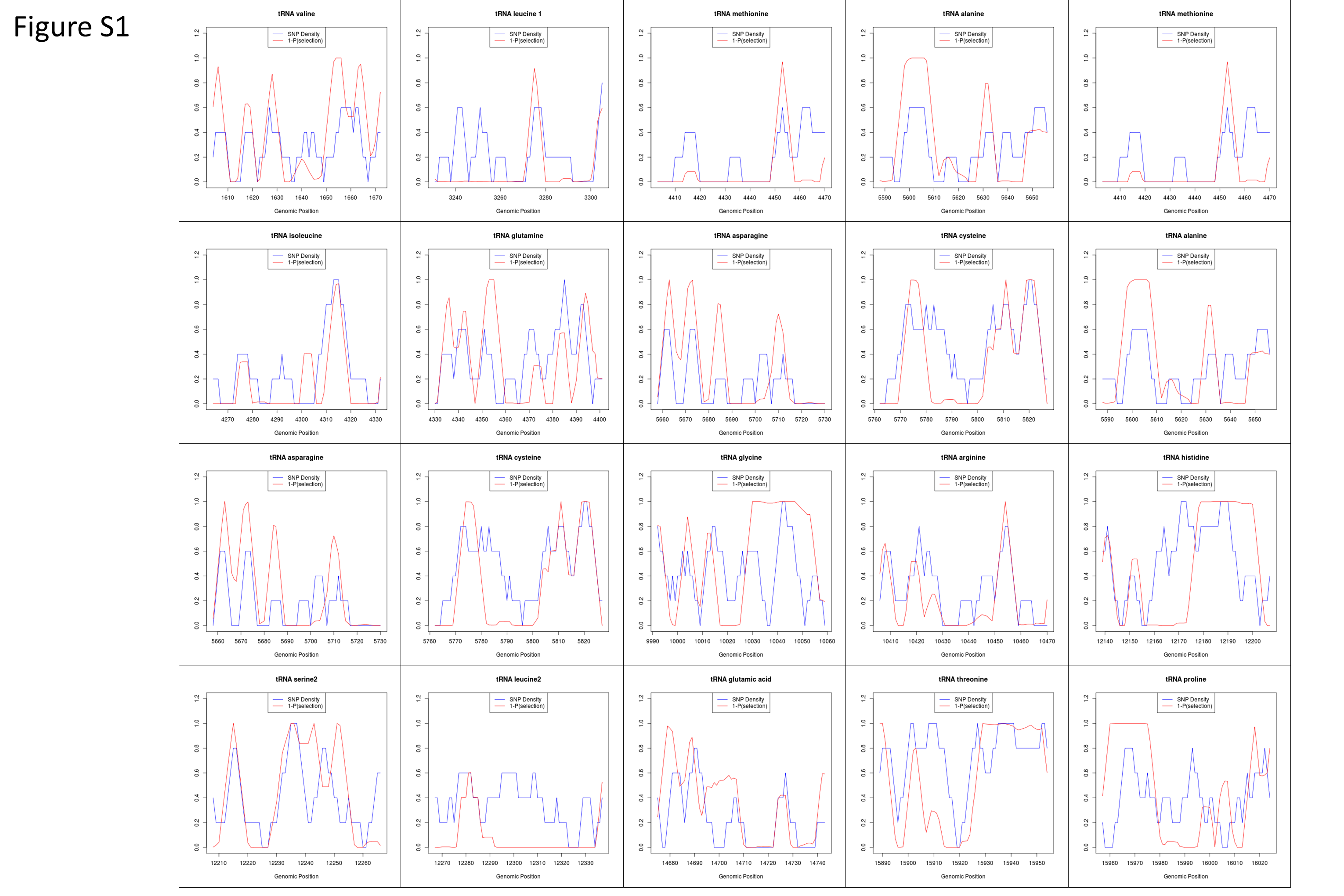

Supplement: Supplementary Data [file supp_evu116_Figure_S1.tif]

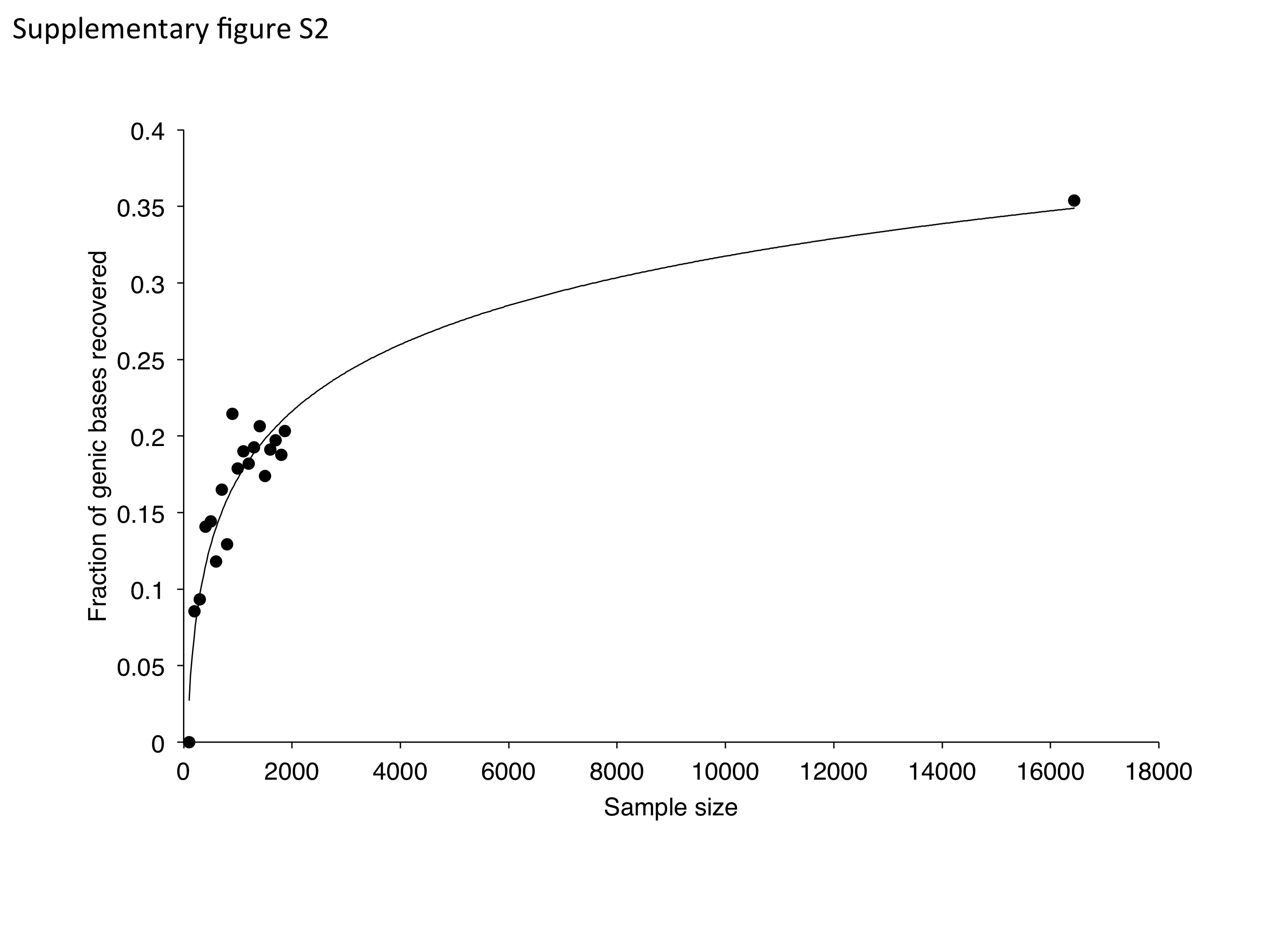

Supplement: Supplementary Data [file supp_evu116_Figure_S2.tif]
